# Post-traumatic stress, awareness, and preparedness among Thai dental students after a century-scale regional earthquake

**DOI:** 10.1371/journal.pone.0341032

**Published:** 2026-02-11

**Authors:** Tanit Arunratanothai, Thanaphum Osathanon, Nareudee Limpuangthip

**Affiliations:** 1 Dental Education Unit, Faculty of Dentistry, Chulalongkorn University, Bangkok, Thailand; 2 Department of Anatomy, Faculty of Dentistry, Center of Excellence for Dental Stem Cell Biology, Chulalongkorn University, Bangkok, Thailand; 3 Department of Prosthodontics, Faculty of Dentistry, Chulalongkorn University, Bangkok, Thailand; Teikyo University Hospital Mizonokuchi, JAPAN

## Abstract

**Objectives:**

To assess responses, post-traumatic stress level, and awareness and preparedness among Thai dental students following the Sagaing earthquake, and to identify the factors associated with their traumatic stress level.

**Methods:**

A questionnaire survey was distributed via Google Form in April 2025 to dental students enrolled at the dental school. The questionnaire consisted of four sections: (1) demographic information; (2) experiences and responses during the earthquake; (3) post-traumatic stress, assessed using the post-earthquake trauma level determination scale; and (4) earthquake awareness and preparedness, assessed using the sustainable scale of earthquake awareness, both using five-point Likert scales. Associations between trauma scores and related variables were analysed using the Wilcoxon Rank Sum or the Kruskal–Wallis test, and multivariable negative binomial regression.

**Results:**

Of 921 students, 287 completed the questionnaire. Initial perceptions during the earthquake were mainly dizziness and fatigue. Immediate responses included drop–cover–hold and stairway evacuation, with most students first grabbing mobile phones, followed by bags and laptops/tablets. Reported reactions focused on concern for loved ones, anxiety about future quakes, and greater appreciation of life and relationships. Multivariable analysis showed that living on the 8th floor or higher was significantly associated with higher post-traumatic stress scores compared to living in houses or lower floors.

**Conclusion:**

The earthquake caused low post-traumatic stress among Thai dental students, though stress was higher in high-rise residents. It increased appreciation of life and relationships. While the faculty response was effective, stronger city- and national-level disaster management is needed for future safety.

## Introduction

Earthquakes are among the most devastating natural disasters, capable of causing extensive loss of life and damage to property and infrastructure. Despite advances in seismic monitoring, accurately predicting the exact timing of earthquakes remains elusive [1]. Countries located along the Pacific Ring of Fire, such as Japan, Indonesia, Chile, the Philippines, and the western part of the United States, are particularly vulnerable and have established comprehensive safety guidelines to mitigate the seismic risks [2–5].

On March 28, 2025, a powerful magnitude moment scale (Mw) 7.7 earthquake struck along Myanmar’s Sagaing Fault [6,7], reaching a maximum Modified Mercalli intensity of IX, making it the strongest recorded earthquake in the country since C.E. 1912. According to the Gutenberg-Richter relationship [8], earthquakes of Mw 7.0 and 8.0 are expected approximately every 60 and 500 years, respectively. A recent seismic hazard study, using an updated earthquake catalogue, identified the Myitkyina-Northern Mandalay segment as the area of highest risk, with a likely event magnitude of Mw 6.4–7.2 [9]. Therefore, the Mw 7.7 event represents a rare, high-impact earthquake, potentially a once-in-a-century occurrence for Southeast Asia. An earthquake with a shallow epicenter (10 km depth) produced seismic waves felt across many countries such as China, Vietnam, and Thailand [6,10]. In Bangkok, the capital of Thailand, tremors were intensified by the city’s soft clay foundation despite the distance from the epicenter, contributing to heightened public concern [11,12].

Earthquakes can disrupt daily life and academic activities and may contribute to psychological distress among affected population, including university students. Dental students are a particular concern due to the demanding nature of their curriculum, which includes lectures, laboratory work, clinical training, and research [13]. In Bangkok, recently affected by a seismic event and home to nearly half of Thailand’s dental schools [14], the earthquake reached a maximum Modified Mercalli intensity of V [15], a level at which shaking is felt by nearly everyone, some individuals become frightened, small objects move or fall from shelves, and some structural effects such as cracked wall may occur, with noticeable movement of trees and bushes [16]. Many institutions are located in high-rise buildings constructed vertically due to land constraints. These buildings serve educational, residential, and clinical functions, increasing student vulnerability during earthquakes. Engaged in hands-on training and patient care, dental students may face elevated physical and emotional risks during seismic events.

According to the Association of Southeast Asian Nations University Network Quality Assurance (AUN-QA) [17], university infrastructures must meet standards of safety, adequacy, and resilience, including preparedness for seismic hazards. Earthquake tremors in educational settings can trigger fear and anxiety, particularly among dental students who spend extended hours in high-rise buildings. Previous studies in students have demonstrated elevated anxiety and post-traumatic stress symptom [18–22]. Moreover, in settings with repeated seismic events, significant stress responses have similarly been documented [23]. Studies in adults and older adults have shown various factors related to increased stress, including age, sex, disaster-related variables such as property damage and loss of loved ones, and inadequate social support [24,25]. Despite evidence of psychological impacts from earthquakes among students, few studies have focused specifically on dental students. Research is particularly limited in contexts with inconsistent earthquake preparedness, such as in Thailand.

There is a lack of evidence on dental students’ psychological impact, awareness, and preparedness related to seismic events in Thailand. Additionally, contextual factors such as perceived structural safety and culturally influenced coping strategies remain underexplored. This earthquake revealed vulnerabilities in both personal resilience and institutional readiness, emphasizing the need to better understand how dental students in low-risk settings perceive and respond to seismic disruptions. Therefore, this study aimed to assess responses, post-traumatic stress level, and awareness and preparedness among Thai dental students following the Sagaing earthquake. The factors associated with their traumatic stress level were also identified.

## Materials and methods

### Study design and participants

This cross-sectional study employed a quantitative approach using a questionnaire survey. The eligible participants were current Thai undergraduate and postgraduate dental students at the Faculty of Dentistry, Chulalongkorn University, during the 2024 academic year. Students who were unwilling to give information were excluded from the study.

The study protocol received ethical approval from the Ethics Committee of the Faculty of Dentistry, Chulalongkorn University (HREC-DCU 2025−062). The study adhered to the principles outlined in the Declaration of Helsinki. Informed consent was obtained from all participants. Consent was documented through completion of the questionnaire, in which participants affirmed their agreement by ticking the designated consent box, in accordance with approval from the institutional ethics committee.

The target population consisted of all undergraduate and postgraduate dental students enrolled at the Faculty of Dentistry, Chulalongkorn University, totaling 921 Thai students (532 undergraduate and 389 postgraduate). The sample size was calculated using Taro Yamane’s formula1967) [26], with a margin of error set at 0.05. Using the formula n=N1+N(e,^2, the sample size was calculated as 9211+921(0.05,^2=278.88. Consequently, the required sample size was 279 participants.

### Translation of post-earthquake trauma questionnaire

The English versions of the post-earthquake trauma level determination scale developed by Tanhan and Kayri (2013) [20] and the sustainable scale of earthquake awareness developed by Genç and Sözen (2021) [27] were translated into Thai following the World Health Organization (WHO) guidelines for the translation and adaptation of instruments [28]. The process included forward translation by two bilingual experts, review by a monolingual Thai group (two dentists and two non-dentists) to address linguistic clarity and cultural relevance, and incorporation of their feedback by the original translators and two additional bilingual reviewers. Subsequently, a professional translator conducted a back-translation into English. Finally, a panel of four bilingual experts reviewed both the original and back-translated versions to ensure conceptual accuracy and equivalence of the Thai versions.

### Data collection

Data was collected using a self-administered questionnaire distributed through an online Google Form (Google LLC, Mountain View, CA, USA). This link was circulated via student representatives from both the undergraduate and postgraduate dental programs. The questionnaire was distributed one week after the mainshock to allow time for the ethical approval process and remained open for a total of four weeks after the mainshock (April 4^th^ to April 25^th^, 2025) to minimize information bias. The questionnaire consisted of four sections as follows (Supporting information).

#### Section 1: Demographic information.

This section collected data on age, sex, academic program, and type of accommodation. Additionally, the location and floor number where participants were staying during the earthquake were recorded. For analytical purposes, building floors were categorized as either high (8^th^ floor and above) or low (below the 8^th^ floor) according to the ministerial regulation issued under the Building Control Act [29].

#### Section 2: Experiences and responses during the earthquake.

The questions included their initial thoughts regarding the cause of the incident, their first actions during the mainshock, and the items taken during the evacuation. Additionally, participants were provided with an open-ended question to describe any other actions or thoughts they had during the event, for example, responses included whether they carried out the drop–cover–hold procedure, a commonly recommended safety measure during earthquakes involving dropping to the ground, seeking cover under stable furniture, and holding on until the tremor subsides.

#### Section 3: Post-traumatic stress level.

The post-earthquake traumatic stress was assessed using the Post-Earthquake Trauma Level Determination Scale [20], which was translated into Thai for this study. The scale consists of 20 items measured on a five-point Likert scale ranging from 1 (strongly disagree) to 5 (strongly agree). It evaluates five dimensions: behavioural problems, excitement limitation, affective symptoms, cognitive structuring, and sleep problems. Eighteen items are positively worded, while the two items reflecting increased behavioural awareness with others and greater life appreciation are negatively worded and were reverse-coded. Individual item scores range from 1 to 5, yielding a total score range of 20–100. Higher post-earthquake trauma scores indicate a greater stress impact resulting from the earthquake [20].

#### Section 4: Earthquake awareness and preparedness.

Earthquake awareness and preparedness were assessed using the Sustainable Scale of Earthquake Awareness [27], which was translated into Thai for this study. The scale comprises 22 items rated on a five-point Likert scale ranging from 1 (totally disagree) to 5 (totally agree). It is divided into three factors: earthquake structure relationship, earthquake preparation application, and earthquake preparedness. Negative items, including worrying about future earthquakes, feeling unsafe, and feeling unprepared about future earthquakes, were reverse-coded. Each item is scored from 1 to 5, resulting in a total score range of 22–110. Higher scores indicate a greater level of earthquake awareness [27].

### Data analyses

Data were analysed using STATA version 17.0 (StataCorp LLC, College Station, TX, USA) with a significance level set at 5%. Descriptive statistics were used to report means and standard deviations (SD), median with the first (Q1) and third quartile (Q3), and frequency distributions (%). Bivariate analyses were conducted to assess associations between the post-earthquake trauma score and each independent variable. The Wilcoxson Rank Sum or Kruskal-Wallis test was applied to assess the associations with categorical independent variables, while Spearman’s rank correlation was used for continuous variables. Subsequently, variables were included in a multivariable negative binomial regression analysis to report incidence rate ratios (IRR) with 95% confidence intervals.

## Results

A total of 287 students participated in this study, representing 31.2% of the target population. Among them, 65.9% were undergraduate students and 34.1% were postgraduate students. The average age of participants was 23.1 ± 3.9 years, ranging from 18 to 41 years. During the earthquake, the majority of respondents were located above the 8^th^ floor, where the clinic and common room are situated, while 20% were on the second floor, where the lecturer rooms are located (Table 1).

**Table 1:** Distribution of participants (n = 287).

Variables	n	%
**Age (years):** (mean ± SD)	23.1 ± 3.9	
**Sex:**		
Male	84	29.3
Female	199	69.3
Not reported	4	1.4
**Academic program:**		
Undergraduate	214	74.6
Postgraduate	72	25.1
Not reported	1	0.3
**Accommodation type:**		
Home	84	29.3
Building, on lower than 8^th^ floor	45	15.7
Building, on 8^th^ floor or above	158	55.0
**Location at the time of earthquake:**		
Underground, ground floor, in vehicle	49	17.1
Building, on 2^nd^ to 7^th^ floor	67	23.3
Building, on 8^th^ floor or above	171	59.6

With regards to experiences during the earthquake (Table 2), the initial perception of the event was most commonly dizziness or lightheadedness, reported by 55.1% of respondents. This was followed by feelings of fatigue or tiredness and recognition of the event as an earthquake, respectively. The most common initial responses were the drop–cover–hold procedure (31.0%) and using the exit stairway (29.3%). Notably, 19.2% continued their usual activities until being informed of the situation. More than three-quarters of students grabbed their mobile phones, followed by bags and laptops, tablets, or other mobile learning devices. Additionally, 23.4% gathered study-related materials such as laboratory and clinical requirement documents and other lab-related items.

**Table 2:** Experiences and responses reported by participants during the earthquake.

Responses	n	%
**A) Initial thoughts regarding the incident cause:**		
Dizziness or lightheadedness	158	55.1
Fatigue or tiredness	42	14.6
Earthquake	40	13.9
Infrastructure-related issues	22	7.7
No noticeable sensation	8	2.8
Companion-induced shaking	5	1.7
Others (gunfire, critical incident, strong wind, rodent, emotional arousal, excessive caffeine intake)	5	1.7
**B) First actions during the mainshock:**		
Drop, cover, and hold	89	31.0
Use the exit stairway	84	29.3
Continue normal activities	55	19.2
Evacuate from the ground floor	28	9.8
Incomplete drop, cover, and hold	7	2.4
Evacuate using a non-designated exit (not the stairway)	7	2.4
Inquire with others (inquire with bystanders, review social media updates)	6	2.1
Do nothing	4	1.4
Others (lower dental chair, secure electronic device)	2	0.7
**C) Items taken during the evacuation:**		
Mobile phone	225	78.4
Bag	147	51.2
Laptop/Tablet/Mobile learning device	127	44.3
Water bottle	53	18.5
Documents	40	13.9
Nothing	17	5.9
Stuff related to clinical/lab works	13	4.5

**Fig 1:**
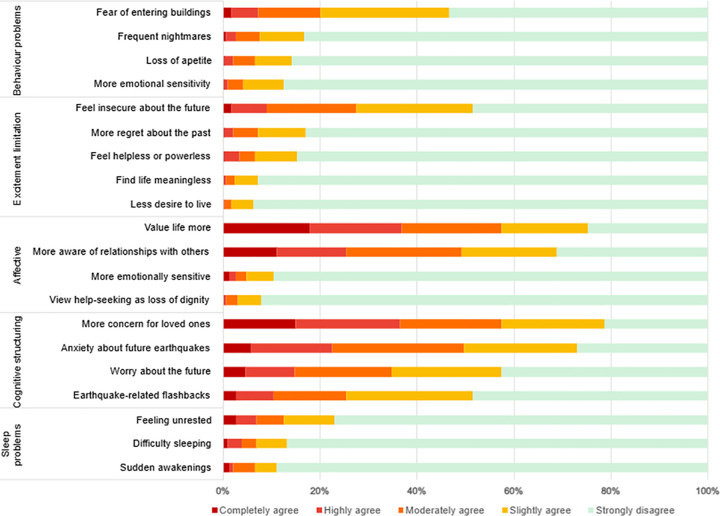
Percentage of participants’ responses on post-traumatic stress level based on post-earthquake trauma level determination scale using a five-point Likert scale.

The mean psychological impact score was 34.265 ± 8.147. Participants most frequently reported agreement with items in the cognitive structuring domain (Fig 1), particularly increased concerns for loved ones (78.7%) and heightened anxiety about future earthquakes (73.2%). This was followed by the affective domain, where participants reported greater life appreciation (75.6%) and increased awareness of their relationships with others (68.3%).

**Fig 2:**
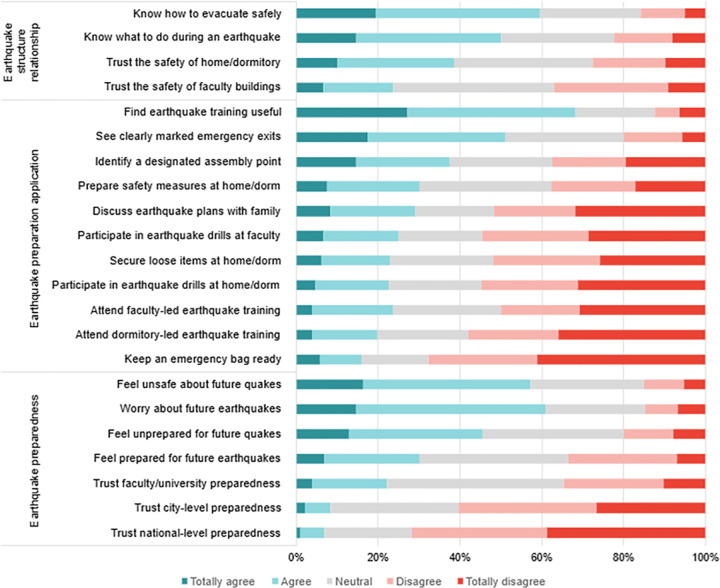
Percentage of participants’ responses on earthquake awareness and preparedness based on the Sustainable Scale of Earthquake Awareness.

Regarding earthquake awareness (Fig 2), the majority of participants expressed worry (61.0%) and a feeling of insecurity (57.1%) about potential future earthquakes. A substantial proportion also reported a lack of trust in preparedness at the national (71.8%) and city (57.1%) levels, while 34.5% indicated distrust in university-level preparedness. Nevertheless, most participants agreed that earthquake training is useful (67.9%).

Bivariate analysis demonstrated that higher stress scores were associated with older age, postgraduate education, and residing in a building, compared to younger individuals, those at the undergraduate educational level, and those residing in a house (Table 3). However, in the multivariable regression analysis, only residing in a building on the 8^th^ floor or higher was significantly associated with increased stress scores when compared to residing in a house (IRR = 1.07, p = 0.039). Although a similar trend was observed for participants residing in buildings below the 8th floor, the association did not reach statistical significance (IRR = 1.08, p = 0.069).

**Table 3:** Bivariate and multivariable analyses of associations between post-earthquake trauma score and related variables.

	Post-earthquake trauma score	Incidence rate ratio (95% CI)
**Total score**	32 (29, 36)	
**Age (years):** (mean ± SD)	Spearman rho = 0.194*	1.01 (0.99, 1.02)
**Gender:**		
Male	32 (29, 38)	1 (reference)
Female	32 (29, 36)	0.98 (0.92, 1.03)
**Academic program:**		
Undergraduate	31 (28, 35)^B^	1 (reference)
Postgraduate	34 (31, 42)^A^	1.06 (0.94, 1.20)
**Accommodation type:**		
Home	31 (28, 34)^B^	1 (reference)^B^
Building, on lower than 8^th^ floor	32 (28, 38)^AB^	1.08 (0.99, 1.17)^B^
Building, on the 8^th^ floor or above	33 (29, 38)^A^	1.07 (1.02, 1.13)^A^
**Place at the earthquake occurred:**		
Underground, Ground, Vehicle	31 (28, 35)	1 (reference)
Building, on the 2^nd^ to 7^th^ floor	33 (28, 36)	1.06 (0.97, 1.15)
Building, on the 8^th^ floor or above	32 (29, 38)	1.04 (0.97, 1.11)

Different capital letters indicate statistically significant differences, determined by Wilcoxon Rank Sum or Kruskal–Wallis test (p < 0.05). An asterisk (*) indicates a significant correlation based on Spearman’s correlation coefficient (p < 0.05).

## Discussion

The present study revealed that the two primary immediate responses of dental students following Sagaing Earthquake were performing the drop–cover–hold procedure and using the exit stairway. Regarding psychological impact, participants commonly reported increased concern for loved ones and anxiety about future earthquakes, increased life appreciation, and heightened awareness of personal relationships. Most participants expressed persistent worry and feelings of insecurity about potential future earthquakes. Residing in buildings on the 8^th^ floor or higher emerged as a significant factor associated with the elevated post-earthquake stress levels compared to the students living in houses or lower-floor buildings.

Although the recent Earthquake that affected Bangkok was classified as a violent Field [10], many dental students initially attributed the sensation to physical exhaustion rather than a seismic event [30]. This misperception reflects the rarity of the seismic events in Thailand and the normalization of mental and physical fatigue within the dental training environment [31]. Once the situation was recognized as an earthquake, students responded appropriately by performing the drop–cover–hold procedure and evacuating via designated fire exits [32,33]. Notably, dental students and personnel in the present context were observed assisting patients during the evacuation, demonstrating both procedural preparedness and professional responsibility.

Most dental students first reached for their mobile phones. This behavior underscores the vital role of mobile technology in emergencies, providing access to communication, real-time information, and practical tools such as flashlights, which contribute to situational awareness and a sense of security during sudden disruptions. A significant proportion of students reported retrieving personal bags and academic devices, such as laptops and tablets, underscoring their concern for preserving coursework, thesis data, and essential materials. This behavior highlights the emotional burden of academic responsibilities and the pressure experienced in professional training.

Despite the severity of the event, most students reported relatively low levels of post-traumatic stress compared to reports from other countries [24,34]. The average post-earthquake trauma score in this study was 34.265 ± 8.147, which falls below the established threshold of 52.385 ± 5.051 for identifying post-earthquake traumatic symptoms [34]. This may reflect cultural characteristics of Thai society, where emotional restraint and collective coping are commonly emphasized in response to adversity. Thai cultural values, such as the pursuit of joy and positive engagement in daily life, may also contribute to psychological resilience [35,36]. In this context, the limited physical damage and absence of casualties, apart from minor damage to decorations, may have contributed to the lower levels of psychological distress, especially when compared to earthquakes that resulted in collapsed buildings and significant loss of life. Nevertheless, the earthquake appeared to serve as a reflective moment. Many students expressed a renewed appreciation for life and recognized the importance of openly expressing care and affection toward loved ones, challenging the reserved norms common in Eastern cultures [37].

A significant association was observed between the increased post-traumatic stress level with the floor level of accommodation, with students residing on higher-floor buildings exhibiting higher stress level. In contrast, the students who were on higher floors at the time of seismic events did not necessarily report increased stress. This differs somewhat from a previous study which reported that individuals situated at greater heights during seismic events may perceive more intense shaking, leading to increased fear and a heightened sense of danger [38]. One explanation may be that a residence is a long-term, personal environment that supports basic daily needs such as sleeping and living [39]. In contrast, faculty buildings are not personally owned spaces and are viewed as places for study and social interaction, where expectations of safety and security may differ [39]. In the present study, post-traumatic stress was assessed after the seismic event while students were staying in their permanent residences, many of which are high-rise apartments where students often live alone, potentially contributing to increased worry and sustained stress responses. These findings underscore the importance of incorporating environmental and structural considerations into mental health preparedness strategies, particularly for populations residing in high-rise accommodations.

In contrast, neither age nor academic programs showed a statistically significant association with post-traumatic stress levels among dental students in the present study. The finding suggests that the psychological impact of the earthquake was relatively uniform across different demographic subgroups, potentially due to a similar level of exposure, shared institutional support, or the cohesive nature of the student environment.

The institutional response was relatively effective. At the faculty level, emergency signage, rapid coordination, and temporary shelters were available within hours [40]. The university issued alerts through mobile applications and deployed structural inspection teams led by engineers, which completed assessments within two days for the faculty [41]. Classes quickly shifted online, and clinical training resumed within five days [42]. These actions provided stability and minimized disruption for students, highlighting the strength of preparedness within the academic structure. These institutional efforts align with the quality assurance principles emphasized by AUN-QA, which advocate for risk preparedness, continuity of academic services, and student-centered resilience during disruptions. Embedding these principles within faculty-level planning not only reinforces institutional credibility but also supports sustainable educational delivery in times of crisis. These institutional strategies reflect the broader context of a VUCA world, where volatility, uncertainty, complexity, and ambiguity increasingly shape both educational and healthcare environments. Integrating disaster preparedness within academic systems thus becomes not only a matter of risk management but also a crucial competency for sustaining professional training under unpredictable disruptions. Nonetheless, areas for improvement include clearer preparedness guidelines, a more audible emergency announcement system, and long-term campus design modifications to allow for safer evacuation in open spaces.

Trust in the city and national-level responses were relatively low. A general statement from the Prime Minister was the only formal communication received during the mainshock, and no real-time emergency alerts were broadcast to personal devices [43,44]. As a result, students turned to social media for updates, which could compromise both clarity and safety. The collapse of a government building far from the epicenter further raised public concern over infrastructure standards [45]. These issues underline the need for robust, transparent systems that not only respond effectively but also build public trust.

This study also demonstrates several methodological strengths. Although the response rate was approximately 31.2%, the final sample size still met the requirements for statistical analysis, as determined by the initial sample size calculation. To our knowledge, this is the first focused exploration of dental students’ experiences of earthquake events in Thailand. However, data were collected solely from a single institution, thus reflecting a specific academic context. The use of self-reported reflections presents inherent limitations due to potential recall bias and shifts in emotional perception over time. Nonetheless, the findings provide valuable insight into how future dental professionals experience disasters, both behaviorally and emotionally. As dizziness or lightheadedness was the most frequently reported symptom, follow-up studies are needed to determine whether these symptoms persist and to evaluate the potential development of post-earthquake dizziness, which is characterized by dizziness, vertigo, imbalance, nausea, and motion sensations [46]. Longitudinal studies examining changes in stress levels following institutional or policy interventions are recommended to support emotional recovery and guide future preparedness efforts.

## Conclusions

The earthquake caused minimal post-traumatic stress among Thai dental students, although residing in high-rise buildings was associated with increased stress levels. The earthquake significantly heightened the students’ awareness of the value of life and the importance of interpersonal relationships. While students demonstrated behavioral preparedness, many appeared emotionally unprepared for future earthquakes. At the institutional level, the faculty response was effective and supportive. However, enhancing disaster management systems at the city and national levels remains crucial to ensure safety, foster confidence, and improve preparedness for future crises.

## Supporting information

S1 File The questionnaire used in this study (English version). (PDF)
