# Supplementary material for: Post-traumatic stress, awareness, and preparedness among Thai dental students after a century-scale regional earthquake
Source: PLoS One. 2026 Feb 11;21(2):e0341032. doi: 10.1371/journal.pone.0341032 (PMC12893554; doi:10.1371/journal.pone.0341032)
Supplement: S1 File — (PDF) [file pone.0341032.s001.pdf]

Evaluation of Thai dental students' traumatic stress and awareness following the Sagaing Earthquakes.

Objective

1. Identify the severity of post-traumatic stress symptoms after earthquakes among dental students at the Faculty of Dentistry, Chulalongkorn University.
2. Identify the level of awareness about earthquake safety measures among dental students at the Faculty of Dentistry, Chulalongkorn University.

Expected Benefit

1. Understand the psychological impact of the earthquake, leading to better mental health and recovery strategies for affected communities.
2. Enhance awareness and preparedness strategies, helping reduce risks and improve response in future earthquake events.

The respondent has read the above information for volunteers. If you choose "Do not consent to participate in the survey," the system will submit your response and end the survey. However, throughout the duration of the survey, if at any point you feel uncomfortable answering, you may stop and withdraw from the project immediately.

☐ I consent to participate in the survey

☐ I do not consent to participate in the survey

Explanation of Consent for the Collection of Personal Data in Accordance with the Personal Data Protection Act B.E. 2562 (2019)

The researcher places a high priority on the privacy and protection of your personal data. In compliance with the Personal Data Protection Act B.E. 2562 (2019), we respectfully seek your consent to collect and use your personal data solely for the purpose of this research study, which aims to investigate post-traumatic stress and earthquake awareness among dental students at the Faculty of Dentistry, Chulalongkorn University. Please be assured that your personal data will be used strictly for the stated objectives and will not be disclosed or used for any other purposes without your explicit consent. If you choose “Do not consent”, your responses will not be recorded, and the survey will be terminated immediately.

☐ Consent

☐ Do not consent

Part 1: Demographic data and status

Please provide the information that best represents your status.

1. Age …… Year
2. Gender ☐ Male ☐ Female
3. Academic Program ☐ Postgraduate student (PG) ☐ Undergraduate student (UG)
4. Primary Type of Residence Before the Earthquake

☐ House ☐ Condominium/Building with fewer than 8 stories

☐ Condominium/Building with 8 stories or more ☐ Other……. ☐ Prefer not to answer

Part 2: Real situation of yourself taken during the earthquake event

Please choose the statement that most accurately reflects your situation/experience.

1. During the earthquake, which floor were you staying on?

☐ Lower ground ☐ On ground ☐ 1st floor ☐ 2nd floor ☐ 3rd floor

☐ 4th floor ☐ 5th floor ☐ 6th floor ☐ 7th floor ☐ 8th floor

☐ 9th floor ☐ 10th floor ☐ 11th floor ☐ 12th floor ☐ 13th floor

☐ 14th floor ☐ 15th floor and above ☐ Prefer not to answer

2. What was your first thought regarding the cause when the incident happened?

☐ Earthquake ☐ Vertigo / Dizziness ☐ Hypoglycemia

☐ Fatigue / Lack of sleep ☐ Others ……. ☐ Prefer not to answer

3. When the main shock of the earthquake happened, what did you do first?

☐ Took cover under a table ☐ Quickly used the emergency stairs

☐ Quickly used the elevator ☐ Continued as usual until someone told me what to do

☐ Others ……. ☐ Prefer not to answer

4. What items did you take with you when evacuating?

☐ Empty-handed / Nothing ☐ Water bottle ☐ Mobile phone ☐ Laptop ipad

☐ Requirement document ☐ Clinic basket ☐ Personal belongings bag

☐ Others ……. ☐ Prefer not to answer

5. If you had any other actions or thoughts, please describe ………………………………

(If you do not wish to respond, please mark "-")

Part 3: Post-Earthquake Trauma Level Determination Scale

The scale consists of 5 domains (20 items) and a five-point Likert structure.

Please choose the statement that most accurately reflects your experience or opinion.

**Behaviour problems**						
Questions	Disagree	Slightly Agree	Moderately Agree	Highly Agree	Completely Agree	Prefer not to answer (N/A)
1. I am experiencing loss of appetite.						
2. I have become a more angry/irritable person.						
3. I am having nightmares.						
4. I cannot enter closed spaces due to the fear of an earthquake.						
**Emotional limitations**						
Questions	Disagree	Slightly Agree	Moderately Agree	Highly Agree	Completely Agree	Prefer not to answer (N/A)
5. I have lost my sense of security about the future.						
6. It feels like life has no meaning anymore.						
7. My desire to live has decreased after what I have experienced.						
8. My regrets about what I have done in my life have increased after the earthquake.						
9. I feel very helpless/powerless.						
**Affective**						
Questions	Disagree	Slightly Agree	Moderately Agree	Highly Agree	Completely Agree	Prefer not to answer (N/A)
10. Needing help hurts my pride.						
11. I have started to pay more attention to my behavior/relationships after the earthquake.						
12. I appreciate the value of my life more.						
13. I have become very emotional/I cry for no reason.						

**Cognitive Restructuring**						
Questions	Disagree	Slightly Agree	Moderately Agree	Highly Agree	Completely Agree	Prefer not to answer (N/A)
14. I worry about my children/parents/friends.						
15. I am anxious with the thought that an earthquake might happen at any moment.						
16. Images of the earthquake come to my mind.						
17. I am worried about the future.						
**Sleep problems**						
Questions	Disagree	Slightly Agree	Moderately Agree	Highly Agree	Completely Agree	Prefer not to answer (N/A)
18. I wake up suddenly from sleep.						
19. I have difficulty falling asleep.						
20. I sleep less.						

Part 4: Sustainable Scale of Earthquake Awareness

The scale consists of 3 domains (22 items) and a five-point Likert structure.

Please choose the statement that most accurately reflects your experience or opinion.

**Earthquake Structure Relationship**						
Questions	Totally Disagree	Disagree	Neutral	Agree	Totally Agree	Prefer not to answer (N/A)
1. In case of an earthquake in the faculty, I have information about what to do.						
2. I know how to evacuate within the school (faculty) in case of danger.						
3. I trust the earthquake resistance of the faculty building I study						
4. I trust the earthquake resistance of the house (dormitory) I live in.						
**Earthquake Preparation Application**						
Questions	Totally Disagree	Disagree	Neutral	Agree	Totally Agree	Prefer not to answer (N/A)
5. In our university, trainings are organized for the probability of an earthquake.						
6. In my dormitory, trainings are organized for the probability of an earthquake.						
7. Emergency exit directions are sufficient in our faculty building.						
8. My family and I sometimes have a meeting on earthquakes.						
9. The university organizes earthquake-related training and meetings.						
10. My dormitory organizes earthquake-related training and meetings.						
11. If meetings about earthquakes (like preparedness or response planning) are helpful.						
12. Have the necessary precautions against the earthquake in the house (in the dormitory).						
13. The earthquake bag in the house (dormitory) is ready.						
14. In the house (dormitory) the items that can be fallen down are fixed to the walls.						

Questions	Totally Disagree	Disagree	Neutral	Agree	Totally Agree	Prefer not to answer (N/A)
15. Assembly point in the chaos that may occur during the earthquake is decided.						
**Earthquake Preparedness**						
Questions	Totally Disagree	Disagree	Neutral	Agree	Totally Agree	Prefer not to answer (N/A)
16. I am ready for a next possible earthquake.						
17. In terms of the whole university, are we prepared for an earthquake?						
18. In terms of the whole city, are we prepared for an earthquake?						
19. In terms of the whole country, are we prepared for an earthquake?						
20. I am worried about the next possible earthquake.						
21. In overall, we are not safe in case of an earthquake.						
22. In overall, we are not prepared for an earthquake.						
